# The function of filensin and phakinin in lens transparency

**Published:** 2008-04-25

**Authors:** Mikako Oka, Hiroaki Kudo, Norio Sugama, Yuko Asami, Makoto Takehana

**Affiliations:** Department of Molecular Function and Physiology, Kyoritsu University of Pharmacy, Tokyo, Japan

## Abstract

**Purpose:**

Beaded filaments are lens cell-specific intermediate filaments composed of two proteins: filensin and phakinin (CP49). Filensin and phakinin are believed to function in the maintenance of lens transparency. To elucidate the function of filensin and phakinin at the molecular level, we examined the degradation of these two proteins in normal and cataractous rat lenses.

**Methods:**

A hereditary cataract model, the Shumiya cataract rat (SCR), was used for these studies. Anti-filensin antibodies were raised against three different regions of the protein, the rod domain, the inner region of the tail domain, and the outer region of the tail domain. Anti-filensin and anti-phakinin antibodies were used to examine the conformation of degradation of filensin and phakinin by western blot analysis and fluorescent immunocytochemistry of cryosectioned lenses.

**Results:**

In the normal lens, filensin was processed from a 94 kDa protein to proteins of 50 kDa and 38 kDa. Similarly, phakinin was processed from a 49 kDa protein to one of 40 kDa. The concentrations of filensin and phakinin in the rat lens cortex fluctuated with age and decreased during cataractogenesis. The 50 kDa form of filensin decreased significantly before opacification. In the normal lens, phakinin, the filensin rod domain, and the filensin inner tail domain localized to membrane lining regions in the shallow cortex and to the central region of the cytoplasm in the deep cortex. The COOH-terminal domain of filensin localized to the membrane lining region in the deep cortex. In pre-cataractous lenses, phakinin and the filensin rod domain localized primarily to the membranes lining the shallow cortex region and were distributed throughout the cytoplasm of lens fiber cells in the deep cortex.

**Conclusions:**

The 50 kDa form of filensin is important for the localization of beaded filaments in lens fiber cells and for lens transparency.

## Introduction

The lens of the eye is composed of two types of cells, epithelial cells, which form a monolayer at the anterior surface of the lens, and lens fiber cells, which originate from epithelial cells and are highly differentiated. Lens fiber cells lack organelles, have lens-specific structures such as gap junctions and beaded filaments, and synthesize lens-specific proteins. Beaded filaments are lens fiber cell-specific intermediate filaments [1] composed of two proteins, filensin [2] and phakinin [3]. Beaded filaments are 15–20 nm in diameter and consist of globular particles with a periodicity of 19–21 nm [4]. Primary amino-acid sequence analysis shows that filensin and phakinin are members of the intermediate filament family of proteins [5-8]. Similar to other intermediate filament proteins: the structure of filensin consists of a head domain, a rod domain that can be divided into three subdomains (1A, 1B, and 2), and a COOH-terminal tail domain. Phakinin is similar in structure to filensin but lacks a COOH-terminal tail domain [9]. Filensin and phakinin copolymerize to form beaded filaments at a stoichiometry of 1:2 to 1:3 both in vivo and in vitro [9,10] but are unable to polymerize with vimentin, another member of the intermediate filament protein family [10].

Beaded filament proteins are found exclusively in the fiber cells of the lens in all vertebrate orders examined [11], which suggests that beaded filaments play a critical role in lens function. Previous studies have shown that the deletion of filensin or phakinin expression in mice by gene targeting causes cataracts and that some forms of hereditary cataracts in humans are caused by mutations of filensin or phakinin [12-17]. These data suggest that beaded filaments play an important role in lens transparency. Full-length filensin is processed into smaller fragments in the normal lens [18], but the significance of its degradation and the function of the degraded protein are unknown.

The Shumiya cataract rat (SCR) hereditary cataract was derived from a congenic line of SHR-fa rats [19], and 66.7% of the animals develop cataracts. Lens opacity first appears in the nuclear and perinuclear regions in 11-week-old SCRs and later develops into a mature cataract. Cataract SCR can be distinguished from normal SCR at six weeks of age by slit-lamp microscopy. The phenotype of the cataract SCR lens manifests as wrinkles along the Y suture. In contrast, the surface of the lens in normal rats and normal SCRs is smooth.

In the current study, we examined normal and pre-cataract SCR lenses at 6 and 10 weeks of age and cataract SCR lenses at 12 weeks of age to determine the role of filensin and phakinin degradation and localization during aging and cataractogenesis.

## Methods

### Animals

Wistar rats and Japanese white rabbits were purchased from Sankyo Labo Service Corporation (Tokyo, Japan). SCRs were bred at the Kyoritsu College of Pharmacy. Six-week-old SCRs were divided into two groups, normal and cataract, based on slit lamp microscopic observation of the Y suture after mydriasis. All animal procedures conformed to the guidelines of the Committee of the Ethics of Animal Experiments at the Kyoritsu University College of Pharmacy (Tokyo, Japan).

### Electrophoresis

For SDS–polyacrylamide gel electrophoresis (SDS–PAGE), rat lenses were homogenized in homogenizing buffer (25 mM Tris-HCl, pH 8.0, 5 mM EDTA, 5 mM EGTA, 1 mM PMSF). The homogenate was centrifuged at 12,000x g at 4 °C for 20 min, and then the pellet was washed twice with the homogenizing buffer. The pellet was extracted with 8 M urea, 5 mM Tris-HCl, pH 8.0, 1 mM EDTA, and 1 mM CaCl_2_ for 20 min. The extract was collected after centrifugation at 13,000x g for 20 min at room temperature. SDS–PAGE was performed using a 7.5% polyacrylamide resolving gel.

### Anti-filensin and anti-phakinin antibodies

Total rat lens RNA was extracted using TRIzol (Invitrogen Corp., Carlsbad, CA) according to the manufacturer’s instructions. Reverse transcriptase polymerase chain reaction (RT–PCR) was performed using the TaKaRa RNA PCR™ Kit (AMV) v3.0 (TaKaRa, Otsu, Japan) and the following oligonucleotide primers: 5′-GAA TTC ATG CTG GAA CGG CTG AAC AA-3′ and 5′-CTC GAG TTA GGA TCC AAG GCT GAG AGA GG-3′ for the filensin rod domain; 5′-GGA CCA TCT GAA ATG ACC TTA-3′ and 5′-GTC ACT AAA CAC ACC CAC TCT G-3′ for the filensin inner-tail domain; 5′-GGA TCC CCT GGA CAG CCC ATG CCA CCT-3′ and 5′-CGG TCG ACT CAA GCC TTG GCA TTT GAG-3′ for the filensin outer-tail domain; and 5′-GGA TCC ATG CCA TTC AGG AAG-3′ and 5′-CTC GAG TTA GTT GTT CTC CTC T-3′ for phakinin. Amplified PCR products were inserted into the pGEX-6P-1 expression vector (GE Healthcare Life Sciences, Buckinghamshire, UK), and recombinant filensin and phakinin peptides were synthesized according to the manufacturer’s instructions.

The recombinant peptides were emulsified with Freünd’s complete adjuvant (ICN Pharmaceuticals, Mesa, CA) and then injected into Japanese white rabbits. The rabbits were given two additional injections of antigen in incomplete Freünd's adjuvant (ICN Pharmaceuticals) at two-week intervals. Antisera were obtained by bleeding from the rabbit ear artery.

### Western blot analysis

Proteins were resolved by SDS–PAGE and then transferred onto a nitrocellulose membrane. The nitrocellulose membrane was blocked with 10% (w/v) skim milk (Wako Pure Chemical Industries Ltd., Osaka, Japan) in TBS (0.9% NaCl, 100 mM Tris-HCl, pH 7.5). Membranes were incubated with anti-filensin, anti-phakinin, and anti-vimentin antibodies followed by horseradish peroxidase-conjugated anti-rabbit antibody (Bio-Rad Laboratories, Hercules, CA). Immunoreactive proteins were visualized with 0.05% diaminobenzidine (Wako Pure Chemical Industries Ltd.) and 0.006% hydrogen peroxide. The data was quantitated using Image J software.

### Semi-quantitative real-time RT–PCR

The primers for quantitative PCR were designed using Primer Express 3.0 software (Applied Biosystems, Foster City, CA) according to the manufacturer’s guidelines. The primers were synthesized by Hokkaido System Science (Sapporo, Japan). The following primer sequences were used: 5′-CCC TGG AAC AAG CTA TTA AGC ATG-3′ and 5′-TTC CGG AGG TTT TCG ATC TG-3′ for filensin; 5′-CTC CAG GCT GAG ACA GAA TCT TTA C-3′ and 5′-TCA TGC CAG TGC TTG GCA T-3′ for phakinin; and 5′-TCC TGT GGC ATC CAT GAA ACT AC-3′ and 5′-AGC ACT GTG TTG GCA TAG AGG TC-3′ for β-actin. Reverse transcription was performed with 1 µg of total RNA and the appropriate reverse primer using the TaKaRa RNA PCR™ Kit (AMV) v3.0. The transcribed cDNA was mixed with SYBR® Green PCR Master Mix (Applied Biosystems), and PCR was performed with the appropriate forward primer using the ABI PRISM 7300 Real-Time PCR System (Applied Biosystems). The reaction conditions were as follows: 95 °C for 5 min; 40 cycles of 94 °C for 1 min, 55 °C for 45 s, and 72 °C for 30 s; and a final extension at 72 °C for 7 min.

### Morphological analysis and immunohistochemistry

Rat lenses were frozen in liquid nitrogen. The frozen lenses were embedded in the OTC compound (VWR International Ltd., Poole, UK) chilled with acetone-liquid carbon dioxide and then cryosectioned into 5 µm sections. The sections were placed on polylysine-coated glass slides and fixed with 4% paraformaldehyde in phosphate-buffered saline (PBS). The sections were blocked with 1% BSA in TBS and then incubated with anti-filensin or anti-phakinin primary antibodies followed by FITC-conjugated anti-rabbit secondary antibody (Kirkegaard & Perry Laboratories Inc., Gaithersburg, MD).

## Results

### Degradation of beaded filament proteins in pre-cataract Shumiya cataract rat lenses

**Figure 1:**
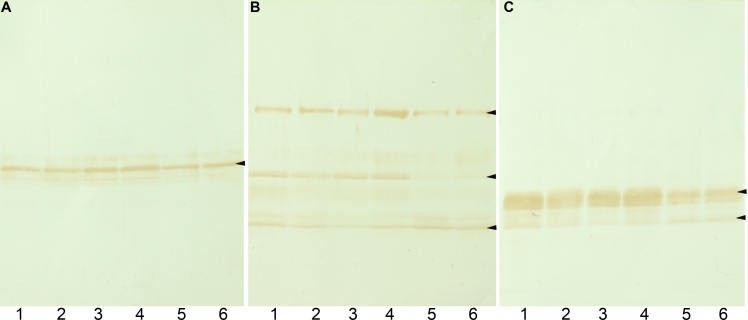
Western blot analysis of intermediate filament proteins in the lens. Western blot analysis of vimentin (**A**), filensin (**B**), and phakinin (**C**) in the lenses of 10-week-old Wistar rats (lanes 1 and 2), normal SCRs (lanes 3 and 4), and pre-cataract SCRs (lanes 5 and 6) is shown. The levels of the 50 kDa form filensin and phakinin in pre-cataract lenses were lower than in control lenses.

Figure 1 shows a western blot analysis of intermediate filament proteins in water-insoluble, urea-soluble fractions of lenses from 10-week-old control Wistar rats (lanes 1, 2), normal SCRs (lanes 3, 4), and pre-cataract SCRs just before opacification (lanes 5, 6). A single major band at 55 kDa was detected by the anti-vimentin antibody (Figure 1A, arrowhead). Three proteins of approximately 94, 50, and 38 kDa were detected by the anti-filensin rod domain antibody (Figure 1B, arrowheads), and the anti-phakinin antibody detected two proteins of approximately 49 and 40 kDa (Figure 1C, arrowheads). The amount of vimentin was the same in all three types of lens (Figure 1A). In contrast, the 50 kDa form of filensin was decreased significantly, and the 38 kDa form was increased in pre-cataract lenses as compared to control lenses. In particular, the 50 kDa form of filensin was markedly decreased (Figure 1B, lanes 5, 6). Similarly, the intact 49 kDa form of phakinin was decreased, and the 40 kDa form was increased in pre-cataract lenses as compared to control lenses (Figure 1C, lanes 5, 6).

### Beaded-filament proteins in the cortices and nuclei of normal and pre-cataract lenses

**Figure 2:**
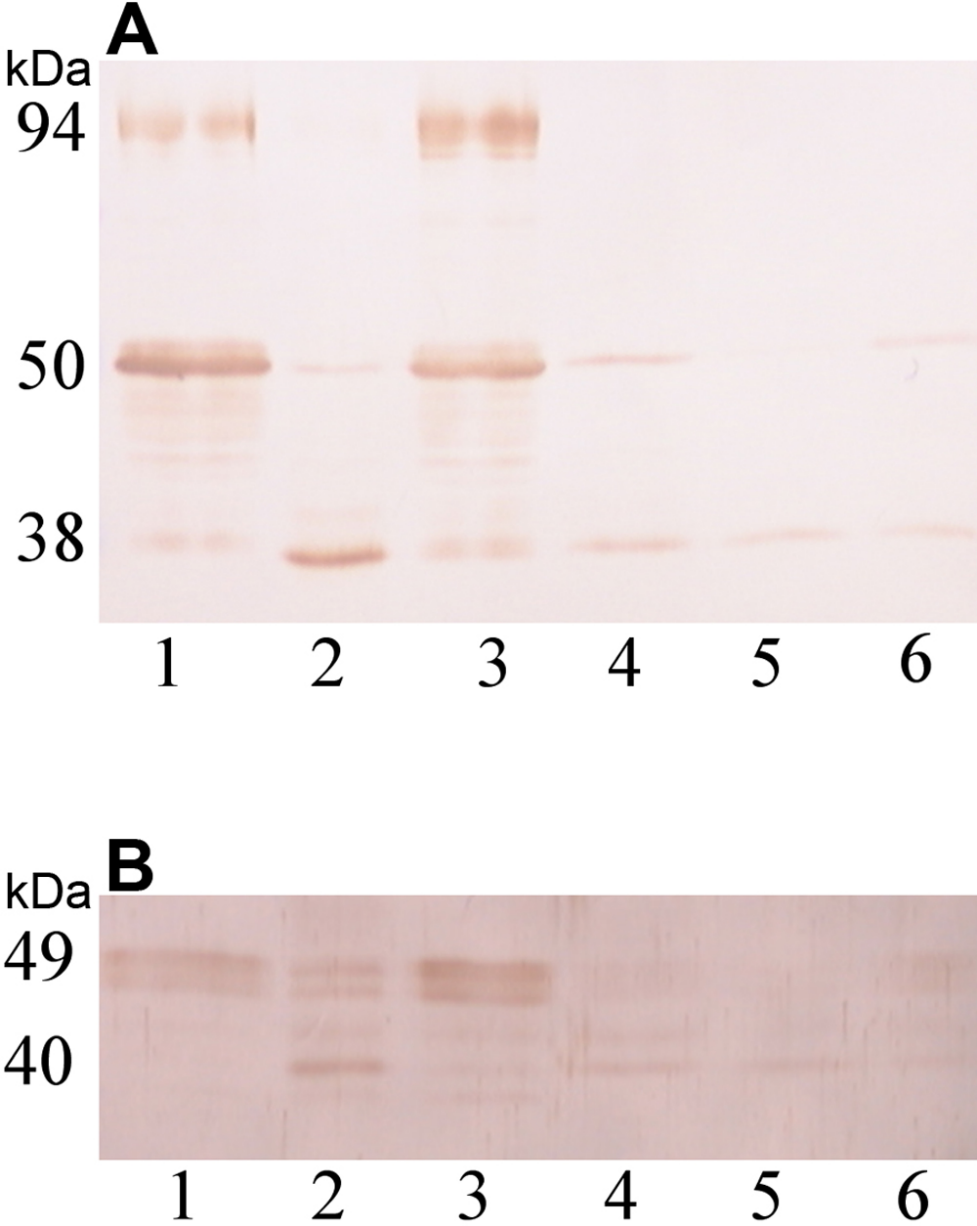
Western blot analysis of filensin and phakinin in the 10-week-old rat lens. SDS–PAGE and western blot analysis of the cortical and nuclear regions of lenses from 10-week-old Wistar rats, pre-cataract SCRs, and normal SCRs using an anti-filensin antibody (**A**) and an anti-phakinin antibody (**B**) are shown. Ten micrograms of protein were loaded in each lane (Lane 1, cortex of Wistar lens; lane 2, cortex of pre-cataract SCR lens; lane 3, cortex of normal SCR lens; lane 4, nucleus of Wistar lens; lane 5, nucleus of pre-cataract SCR lens; lane 6, nucleus of normal SCR lens). The levels of filensin and phakinin in the nuclear regions were lower in 10-week-old lenses than in 6-week-old lenses. The 94 kDa form of filensin was absent from the cortex of the pre-cataract SCR lens. The 50 kDa form of filensin was decreased and the 38 kDa form was increased in the cortices of pre-cataract lenses as compared to Wistar lenses.

To clarify the functions of filensin and phakinin in the maintenance of lens transparency, changes in the protein levels of filensin and phakinin in the cortex and nuclear regions of the lens were examined by SDS–PAGE and western blot. The cortical and nuclear regions of Wistar, pre-cataract SCR, and normal SCR lenses were separated, and the levels of filensin and phakinin were examined at the age of 6, 10, and 12 weeks. Cataract SCRs can be distinguished from normal SCRs at six weeks of age, and they develop cataracts at 11 weeks of age. The levels of the 94 kDa form of filensin in the cortical regions of normal, cataract SCR, and normal SCR lenses were similar at six weeks of age. At 10 and 12 weeks of age, there was less 94 kDa filensin in the cortical regions of cataract SCR lenses than in Wistar and normal SCR lenses. The 94 kDa form of filensin was absent from the nuclear regions of Wistar, cataract SCR, and normal SCR lenses at 6, 10, and 12 weeks of age. The level of the 50 kDa form of filensin was slightly lower at six weeks of age and markedly decreased at 10 and 12 weeks of age in the cortex of the cataract SCR lens as compared to Wistar and normal SCR lenses. Overall, the level of 50 kDa filensin was lower in nuclear regions than in cortical regions, and it was absent from the nuclear regions of cataract SCR lenses at 6, 10, and 12 weeks of age. The cortical regions of 10- and 12-week-old cataract SCR lenses contained more 38 kDa filensin than Wistar and normal SCR lenses (Figure 2A).

In six-week-old cataract SCRs, the 49 kDa form of phakinin was slightly degraded and the 40 kDa form appeared in the cortex of the lens (data not shown). At the age of 10 (Figure 2B) and 12 weeks, most of the 49 kDa form of phakinin was degraded in cataract SCR lenses. Figure 2 shows a western blot of filensin and phakinin in the cortical and nuclear regions of 10-week-old Wistar, cataract SCR, and normal SCR lenses. In cataract SCR lenses, both the 50 kDa form of filensin and the 49 kDa form of phakinin were degraded at 10 weeks of age

**Figure 3:**
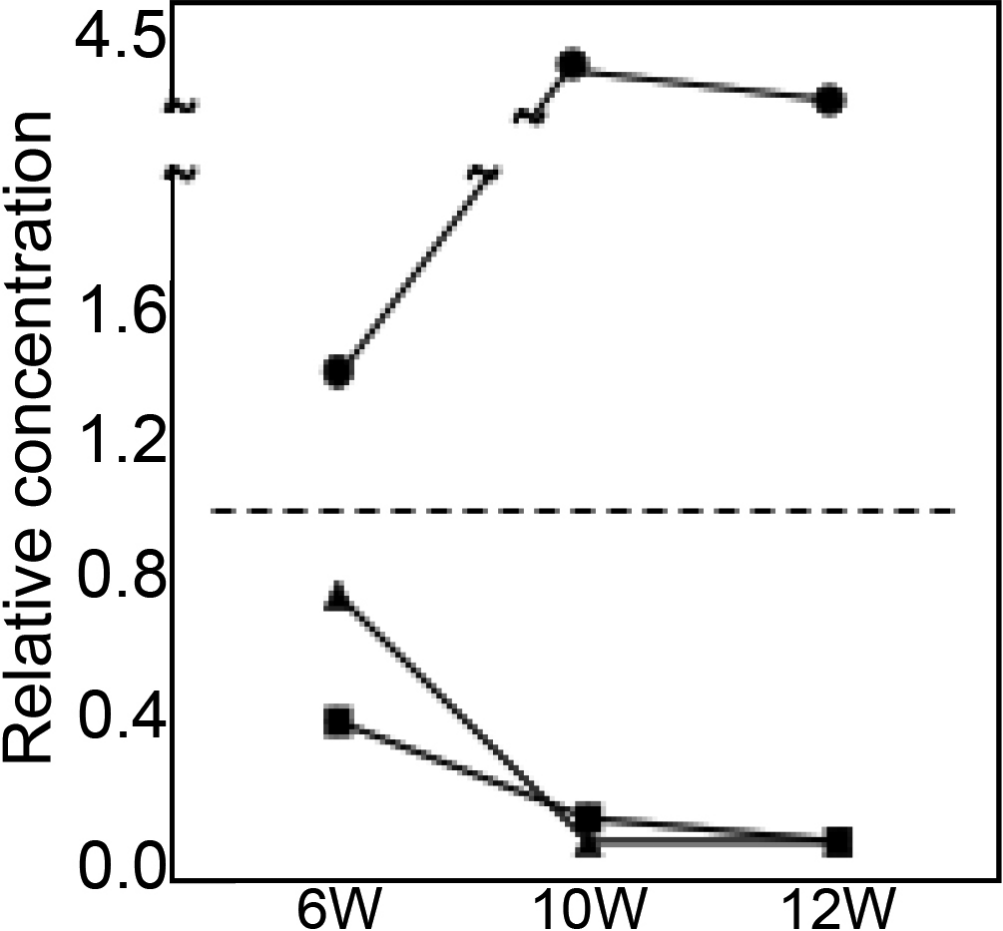
The relative amounts of the 94 kDa, 50 kDa, and 38 kDa forms of filensin in the rat lens. The amount of each of the molecular weight forms of filensin in cataract SCR and Wistar lenses was analyzed by western blot and then quantitated by scanning. The amount of the 94 kDa, 50 kDa, and 38 kDa forms of filensin in cataract SCR lenses is shown relative to Wistar lenses. Triangle, 94 kDa filensin; Square, 50 kDa filensin; Circle, 38 kDa filensin.

The relative intensity of each molecular weight form of filensin in the cortical regions of cataract SCR and Wistar lenses was quantitated using densitometry, and the results are presented in Figure 3. The level of 94 kDa filensin was lower in SCR lenses than in Wistar lenses at 6 weeks of age. At 10 and 12 weeks of age, the 94 kDa form of filensin was nearly undetectable. The level of 50 kDa filensin in cataract SCR lenses was approximately half the level in Wistar lenses at six weeks of age and was undetectable at 10 and 12 weeks of age. In contrast, cataract SCR lenses contained higher levels of 38 kDa filensin than Wistar lenses at 10 and 12 weeks of age. The amount of 38 kDa filensin in SCR lenses was more than fourfold higher than in Wistar lenses due largely to the very low level of 38 kDa filensin in Wistar lenses.

**Figure 4:**
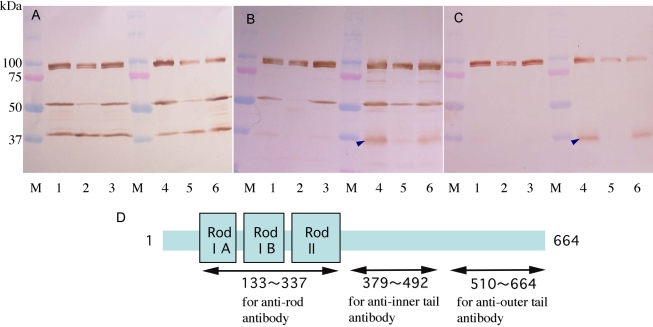
Western blot analysis of filensin. Western blot analysis was performed using antibodies directed against the filensin rod domain (**A**), the inner tail domain (**B**), and the outer tail domain (**C**). Water-soluble fractions of 10-week-old Wistar (lanes 1), pre-cataract SCR (lanes 2), and normal SCR (lanes 3) lenses; water-insoluble and urea-soluble fractions of 10-week-old Wistar (lanes 4), pre-cataract SCR (lanes 5), and normal SCR (lanes 6) lenses. Arrowheads in **B** and **C** indicate the 36 kDa filensin peptide. The schematic representation of the filensin rod and tail domains (**D**) shows the predicted antigen sites (arrows) and cleavage sites (arrowheads).

To investigate the degradation of filensin in more detail, western blot analysis was performed using antibodies specific for the inner (Figure 4B) and outer regions (Figure 4C) of the filensin tail domain. The anti-filensin rod domain antibody recognized 94 kDa, 50 kDa, and 38 kDa forms of filensin, and the levels of 94 kDa and 50 kDa filensin were lower in water-soluble fractions than in water-insoluble and urea-soluble fractions of pre-cataract SCR lenses (Figure 4A). The filensin inner tail domain antibody recognized the 94 kDa and 50 kDa forms of filensin but not the 38 kDa form of the protein. It also recognized a peptide of 36 kDa in water-insoluble and urea-soluble fractions (Figure 4B, arrowhead). In pre-cataract SCR lenses, the 94 kDa and 50 kDa forms of filensin were lower than in control lenses and the 36 kDa peptide was undetectable (Figure 4B, lane 5). The anti-filensin outer tail domain antibody recognized the 94 kDa form of filensin and the 36 kDa peptide but not the 50 kDa or 38 kDa forms of filensin. In pre-cataract SCR lenses, there was less 94 kDa filensin than in Wistar and normal SCR lenses and the 36 kDa peptide (arrowhead) was undetectable (Figure 4C, lane 5). These results suggested that the 94 kDa form of filensin is cleaved into rod and tail domains of 50 kDa and 36 kDa, respectively, and that the 50 kDa filensin rod domain is further cleaved to generate a 38 kDa peptide. The predicted domain structure of rat filensin is shown in Figure 4D, and the positions of the amino acids that comprise the outer and inner tail domain antigens are indicated along with the predicted filensin cleavage sites.

### Histological analysis of rat lens fiber cells and the localization of filensin and phakinin in the rat cortical region

**Figure 5:**
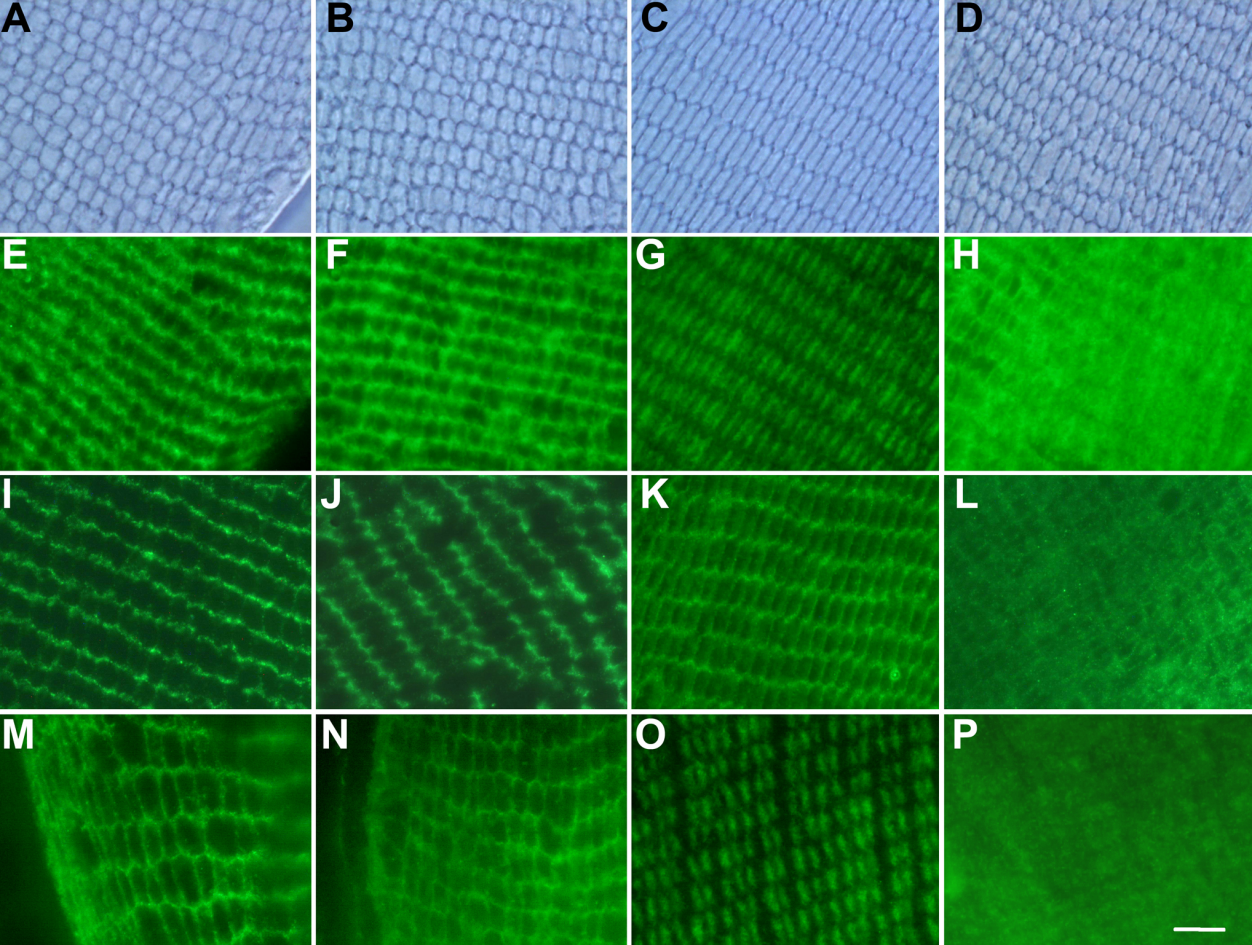
Localization of filensin and phakinin in the 10-week-old rat lens. The localization of filensin in 10-week-old rat lenses was examined by phase-contrast microscopy (**A**-**D**) and fluorescence immunochemistry using antibodies directed against the filensin rod domain (**E**-**H**), filensin outer tail domain (**I**-**L**), and phakinin (**M**-**P**). (**A**, **E**, **I**, and **M**) shallow cortex of the Wistar lens; (**B**, **F**, **J**, and **N**) shallow cortex of the pre-cataract SCR lens; (**C**, **G**, **K**, and **O**) deep cortex of the Wistar lens; (**D**, **H**, **L** and **P**) deep cortex of the SCR lens. The anti-filensin rod domain antibody localized to the membrane lining regions in the shallow cortices of Wistar and pre-cataract SCR lenses (**E** and **F**) and to the central region of the cytoplasm in the deep cortex of the Wistar lens (**G**). The anti-filensin rod domain antibody exhibited a diffuse staining pattern in the deep cortex of the cataract SCR lens (**H**). The anti-filensin outer tail domain antibody localized to the membrane lining region of the shallow cortex of the Wistar (**I**) and pre-cataract SCR lens (**J**) as well as the deep cortex of the Wistar lens (**K**). This antibody exhibited a diffuse staining pattern in the deep cortex of the pre-cataract SCR lens. The localization of phakinin (**M**-**P**) was similar to that of the filensin rod domain (**E**-**H**). Scale bar, 10 µm.

The morphology of lens cells and the localization of filensin in the shallow and deep cortices of the rat lens were examined in cross-sectional tissue samples using the anti-filensin rod domain antibody (Figure 5). Normal (Figure 5A,C,E,G) and pre-cataract SCR lenses (Figure 5B,D,F,H) at 10 weeks of age were incubated with anti-filensin rod domain antibody, and then the sections were viewed by phase microscopy (Figure 5A-D) and fluorescence-microscopy (Figure 5E-H). In pre-cataract SCR lenses, phase microscopy revealed the presence of vacuoles in the lens fiber cells and the cellular architecture was disordered (Figure 5B). In normal SCR lenses, the cells were more regular in structure, but some cells were larger than others and vacuoles were occasionally observed (not shown). Fluorescence microscopy revealed a pattern of fluorescence that corresponded to the outer edges of the lens fiber cells, which indicated that filensin localizes to the membrane lining regions in normal lenses. There were no significant differences in the localization of filensin in the shallow cortices of Wistar, cataract SCR (Figures 5E,F), and normal SCR (data not shown) lenses. The cells of the normal rat lens were smaller in the deep cortex than in the shallow cortex (Figures 5A,C). In the deep cortex of the Wistar lens, anti-filensin rod domain antibodies localized to the central region of the cytoplasm in lens fiber cells (Figures 5C,G) whereas in pre-cataract SCR lenses, filensin appeared to be distributed throughout the fiber cell (Figures 5D,H). We observed a similar pattern of filensin distribution using the filensin inner tail domain antibody (not shown).

We also examined the localization of filensin using a COOH-terminal antibody directed against the outer region of the filensin tail domain. Western blot analysis indicated that the filensin outer tail domain antibody recognizes the 94 kDa form of filensin but not the 50 kDa or 38 kDa forms of the protein. In the shallow cortices of Wistar and pre-cataract SCR lenses, anti-filensin outer tail domain antibodies localized to the subcellular membrane regions (Figures 5I,J, respectively). In the deep cortex of the Wistar lens, the antibody localized to subcellular membrane regions (Figure 5K) whereas in pre-cataract SCR lenses, the anti-filensin outer tail antibody was distributed throughout the cytoplasm of lens fiber cells (Figure 5L).

When we performed a similar type of analysis using anti-phakinin antibodies, we observed that phakinin localizes to subcellular membrane regions in the shallow cortices of both Wistar and pre-cataract SCR lenses (Figure 5M,N). In the deep cortex of the Wistar lens, anti-phakinin antibodies localized to the central region of the lens fiber cell cytoplasm (Figure 5O). In pre-cataract SCR lenses, phakinin was distributed throughout the fiber cell (Figure 5P). These results indicated that the localization of phakinin is similar to filensin.

### Semi-quantitative analysis of filensin and phakinin expression

**Figure 6:**
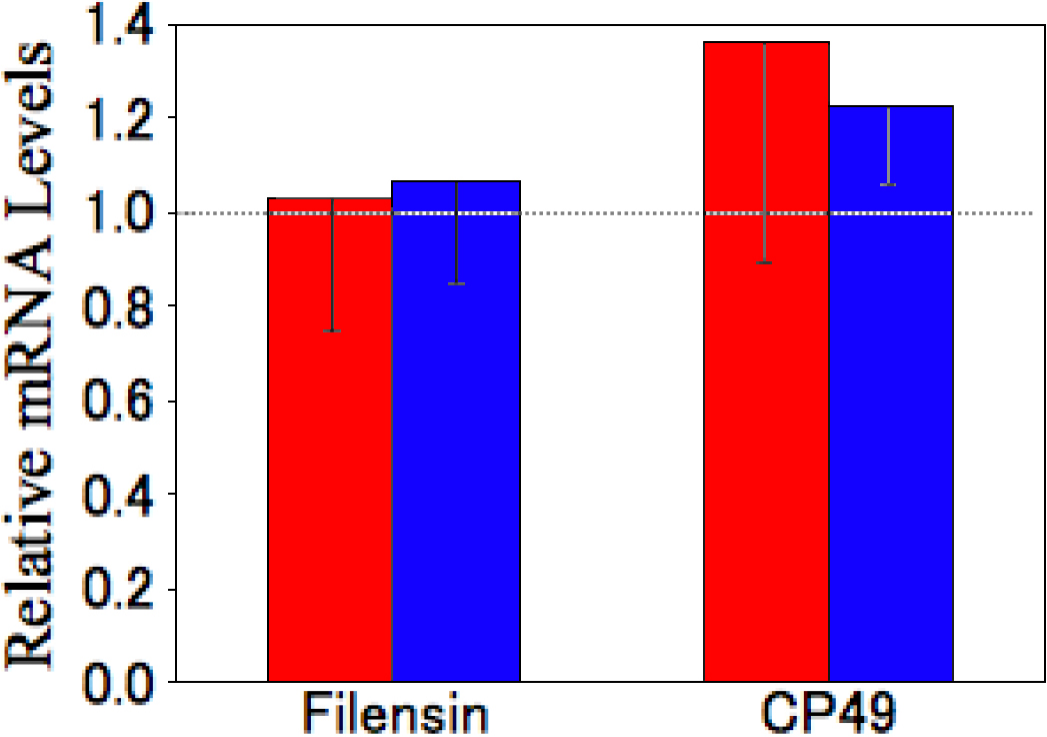
Semi-quantitative analysis of filensin and phakinin mRNAs. The amounts of filensin and phakinin mRNA in pre-cataract SCR and normal SCR lenses were analyzed by semi-quantitative PCR. The data are presented relative to the levels in Wistar rat lenses (1.0). Solid bars represent pre-cataract SCR lenses, open bars represent normal SCR lenses. mRNA levels were normalized to the level of cytoplasmic beta-actin mRNA. Data represents the means and standard deviation of four independent experiments.

Semi-quantitative RT–PCR was used to investigate the levels of filensin and phakinin mRNA in the lens using the expression of beta-actin as a positive control (Figure 6). No significant differences in the mRNA levels of filensin and phakinin were observed among Wistar, cataract SCR, or normal SCR lenses.

## Discussion

### Degradation of filensin

Filensin is a 94 kDa intermediate filament protein and a component of the beaded filament of lens fiber cells. In this report, we have shown that filensin is processed into two smaller molecular weight proteins of 50 and 38 kDa in the normal rat lens. It has been reported that filensin is processed in a similar manner in normal bovine lenses [18]. Using antibodies specific for different domains of the protein, we have shown that both the 50 kDa and 38 kDa forms of filensin contain the rod domain and the 50 kDa form of filensin contains a portion of the tail domain that is adjacent to the rod domain.

Presumably, the 50 kDa, 38 kDa, and 36 kDa forms of filensin are products of proteolytic cleavage; however, there are few known proteinases in the lens. Calpain is a calcium-activated cysteine protease, and many isoforms of calpain have been identified in the lens including calpain 2, calpain 3, calpain 10, LP82, and LP85 in the lens [20-24]. However, filensin is processed into 50 kDa, 38 kDa, and 36 kDa forms in the normal lens in which calcium levels are very low, suggesting that the filensin cleavage products are not generated by calpain. Calpains are activated by calcium, and calcium levels rise in the lens after the onset of cataractogenesis. In the SCR lens, calpain has been suggested to be the major proteinase involved in cataractogenesis [25]. Crystallins are also believed to be targets of calpain-mediated degradation, and insoluble aggregates of truncated crystallin have been proposed to play a role in the formation of cataracts [26].

Additional candidate proteinases that are present in the lens are caspase and the proteasome. Caspase activates a DNase that is involved in lens development and nuclear degeneration [27], and proteasomes are involved in the complete digestion of cellular proteins [28]. Thus, neither caspase nor the proteasome are likely candidates for the proteinase that cleave filensin.

In the pre-cataract SCR lens, the degradation of filensin and phakinin was accelerated compared to normal rat lenses. The mRNA sequences of filensin and phakinin are the same in Wistar, cataract SCR, and normal SCR lenses (data not shown). Furthermore, we performed a semi-quantitative analysis of filensin and phakinin mRNA levels and found that they are similar in all three types of lens (Figure 6). This data indicates that the degradation of filensin and phakinin in pre-cataract SCR lenses is not caused by decreased transcription or conformation changes brought about by alterations in the primary amino-acid sequences of the proteins. Thus, an unidentified protease and/or peptidase is likely to be involved in the degradation of filensin and phakinin. Presumably, different proteases are involved in generating the 50 and 38 kDa forms of filensin. Furthermore, generation of the 50 kDa form of filensin may involve a different enzyme in cataract SCRs than in normal rats.

### Polymerization of filensin and phakinin and the role of beaded filaments

Filensin and phakinin copolymerize into beaded filaments at a molar ratio of 1:2 to 1:3 [9,10]. Structural studies have suggested that beaded filaments are 15–20 nm in diameter and that the “beads” have a periodicity of 19–21 nm [4]. However, a detailed molecular structure of beaded filaments has yet to be reported. It has been suggested that the filensin tail domain comprises the bead portion of the beaded filament [4]. It has also been reported that the bead portion includes a third component, α-crystallin [10]. Further complicating the issue, other groups have suggested that the beaded filament is smooth rather than beaded [9]. Thus, a clear picture of the structure of beaded filaments is still emerging. In addition, all of the molecular components of beaded filaments have not been identified. In the current study, we have shown that in the normal lens, the 94 kDa form of filensin is degraded and that in the deep cortex, the filensin COOH-terminal tail domain localizes to membrane lining regions whereas the filensin rod domain and phakinin localize to the central regions of lens fiber cells. These results suggest that the COOH-terminal region of the tail domain of filensin is not essential for the polymerization of beaded filaments.

In the normal rat lens, the filensin rod domain and phakinin localized to the membrane lining region in shallow cortex cells and to the central region of the cytoplasm in deep cortex cells. These results are in agreement with previous studies of the mouse lens [29]. In cells of the deep cortex, the filensin tail domain localized to subcellular membranes, but the filensin rod domain did not. As beaded filaments are composed of both filensin and phakinin, the polymerized form of beaded filaments should localize to subcellular membranes in the shallow cortex and to the central region of lens fiber cells in the deep cortex. The physiologic significance of the transition from membrane lining regions to the central region of the fiber cell during fiber cell differentiation is unknown. It is possible that the beaded filament is necessary for the formation of adhesion structures in young elongated fiber cells or that the structure that underlies the fiber cell membrane may be necessary for beaded filament polymerization in the shallow cortex of the lens. Polymerized beaded filaments separate from the cell membrane and localize to the center of fiber cells where they may play an important role in the maintenance of lens transparency. In the deep cortex of pre-cataract SCR lenses, filensin and phakinin were distributed throughout the cytoplasm. This result indicates that filensin and phakinin are not incorporated into beaded filaments in the deep cortex of pre-cataract SCR lenses and that polymerized beaded filaments in deep cortex cells are important for lens transparency.

The 50 kDa form of filensin was markedly decreased in pre-cataract and cataract lenses, and the localization of filensin was disordered in the deep cortex of pre-cataract lenses. These results indicate that the 50 kDa form of filensin is important for the polymerization of beaded filaments and lens transparency. The 38 kDa form of filensin was detected at very low levels in the lens cortices of young animals, which indicates that the 38 kDa form of filensin is not required for beaded filament polymerization. In conclusion, we have shown that polymerized beaded filaments are important for lens transparency and that the 50 kDa form of filensin may be a critical component of beaded filaments.
